# Objective Detection of the Speech Frequency Following Response (sFFR): A Comparison of Two Methods

**DOI:** 10.3390/audiolres12010010

**Published:** 2022-01-28

**Authors:** Fan-Yin Cheng, Spencer Smith

**Affiliations:** Department of Speech, Language and Hearing Sciences, The University of Texas at Austin, Austin, TX 78712, USA; fanyin.cheng@utexas.edu

**Keywords:** frequency following response (FFR), auditory electrophysiology, Hotelling’s T^2^ test, F-test, objective detection

## Abstract

Speech frequency following responses (sFFRs) are increasingly used in translational auditory research. Statistically-based automated sFFR detection could aid response identification and provide a basis for stopping rules when recording responses in clinical and/or research applications. In this brief report, sFFRs were measured from 18 normal hearing adult listeners in quiet and speech-shaped noise. Two statistically-based automated response detection methods, the F-test and Hotelling’s T^2^ (HT^2^) test, were compared based on detection accuracy and test time. Similar detection accuracy across statistical tests and conditions was observed, although the HT^2^ test time was less variable. These findings suggest that automated sFFR detection is robust for responses recorded in quiet and speech-shaped noise using either the F-test or HT^2^ test. Future studies evaluating test performance with different stimuli and maskers are warranted to determine if the interchangeability of test performance extends to these conditions.

## 1. Introduction

Neural encoding of speech features can be noninvasively assessed using the speech frequency-following response (sFFR). Over the past two decades, the sFFR has been used in a variety of research studies examining: effects of auditory expertise and deprivation on speech feature encoding [1,2,3,4,5,6,7]; neural representation of speech in adverse listening conditions [8,9,10]; objective hearing aid fitting [11]; bimodal hearing assessment [12,13,14]; and auditory development [15], to name a few examples. Despite the broad use of this technique, few studies, e.g., [11,16,17,18] have examined statistically-based automated response detection of sFFRs. This consideration is important in both clinical and research domains, as automated detection would aid in objective response identification and would provide a statistical basis for stopping rules when recording sFFRs.

This brief report examined accuracy and test time required to objectively detect sFFRs in quiet and noise using F- and Hotelling’s T^2^ (HT^2^) tests in the frequency domain (see [19,20] for detailed reviews of statistical techniques for objective response detection). The F-test calculates an F ratio of signal power at a single frequency of interest (e.g., the fundamental frequency, F0) to the mean power of adjacent frequency bins in which no response components are expected. This approach is particularly well-suited for detecting auditory steady state responses in objective audiometry, e.g., [21,22]. The HT^2^ test is a multivariate analysis in which mean differences of measured and hypothesized values are tested for significance using multiple sFFR features (e.g., F0 and harmonics). The HT^2^ test is thus ideal for automated sFFR detection, as it analyzes information at multiple frequencies to determine if a response is present [6,23,24]. Further, this test may be more powerful in conditions where individual components are degraded (e.g., when the sFFR is measured in background noise).

The purpose of this study was to objectively detect sFFR responses using both F- and HT^2^ tests and to compare detection accuracies and test times for both approaches. We hypothesized that the HT^2^ test would have better detection accuracy and shorter time-to-detect because it uses more information contained in the sFFR than the F-test.

## 2. Materials and Methods

### 2.1. Participants

The University of Texas at Austin approved the methods described in this study. Participants were 18 adults (10 females, age range 20–32). None reported a history of otopathology, neuropathology, or significant noise exposure. All passed otoscopy, tympanometry, and pure tone audiometry screening.

### 2.2. Stimulus and Recording Procedures

sFFRs were elicited diotically using a 170-ms/da/speech token (F0 = 100 Hz). Stimuli were presented in alternating polarity at 70 dB SPL through electromagnetically shielded ER-3C insert earphones at a rate of 4.3 Hz [13]. In the noise condition, continuous speech-shaped noise was presented diotically at 65 dB SPL. sFFRs were recorded with a Cz-C7 single channel montage for 2000 stimulus repetitions using a Neuroscan SynAmps2 system (Compumedics Neuroscan; Charlotte, NC, USA). Responses were artifact rejected at ±30 µV and filtered from 70–2000 Hz using Curry 8 software. Further analyses were conducted offline in MATLAB (The Mathworks, Natick, MA, USA).

### 2.3. Automatic Response Detection

F-statistic and HT^2^ analyses were performed on frequency-domain data extracted from the 60–180 ms epoch (i.e., steady-state vowel portion) of the “added” sFFR, which biases the responses to reflect speech envelope features (see [25]). The F-statistic approach was implemented on the spectrum of the cumulative average sFFR waveform each time a sweep was added to the average, as described by Picton and colleagues (2003) [20]:

$$F=B\left(x_{F0}^{2}\;+\;y_{F0}^{2}\right)/\overset{F0+5+B/2}{\underset{\begin{array}{c} j=F0-5-B/2\\ j\neq F0 \end{array}}{\sum }}\left(x_{j}^{2}\;+\;y_{j}^{2}\right)$$

where F0 is the fundamental frequency (averaged across 99–101 Hz bins), *j* is the frequency of an adjacent noise bin, *B* is the number of noise bins, and *x* and *y* are amplitude and phase components of a specified frequency bin. In the present experiment, *B* = 18, and the upper and lower samples of noise began at F0 ± 5 Hz, respectively. The respective degrees of freedom were 6 and 36 for the numerator and denominator [19,20].

The HT^2^ test was also implemented as described by Picton and colleagues (2003) using the equation [20]:

$$T^{2}=N(\bar{x}\;-\;\mu_{0})S^{-1}(\bar{x}\;-\;\mu_{0})’$$

where N  is the number of sweeps, $\bar{x}$ is a vector of measured dependent variable means with length L, μ_0_ is a vector of hypothesized means with length L, and *S*^−1^ is an inverse covariance matrix of N×L. In the present experiment, both amplitude and phase components from F0, H2, H3, and H4 were used as dependent variables in the HT^2^ test; thus, L=8. Because the data are circular, the vector of hypothesized means (i.e., the expected outcome if a response were not present) consists of zeros. *T*^2^ was converted to an F-statistic using the equation:

$$F=\frac{N-L}{L\left(N-1\right)}T^{2}\tilde{\;}{\;}_{F_{df1,df2^{\;}}}$$

where $df_{1}=L\;$ and $df_{2}=N-L\;.$

**Figure 1:**
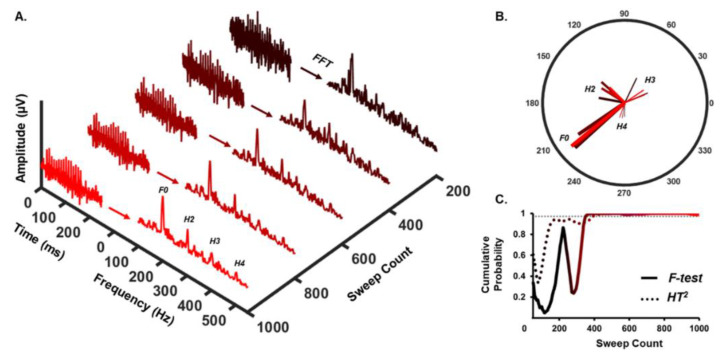
Automatic Response Detection Approach. (**A**) Average sFFR waveforms collected in quiet (left) and their corresponding spectra (right) are shown as a function of sweep count for a single participant (black-to-red color transition indicates an increase in cumulative sweeps contributing to the average from 200–1000). Frequency bins of interest for the F-statistic (F0 only) and HT2 test (F0-H4) are labeled on the final spectrum. (**B**) Amplitude and phase components are extracted from each frequency bin of interest and used as dependent variables for statistical tests. Note that each vector maintains the color-coding denoting sweep count from panel A. Note also that the F-test only uses magnitude and phase from the F0 vector, whereas HT2 uses magnitude and phase components from all vectors shown. (**C**) Cumulative probability functions are plotted as a function of sweep count for each test with 99% confidence denoted by the gray dotted line. In this single-subject example, F- and HT2 tests perform similarly, as an sFFR is detected with >99% confidence between 380–400 sweeps with each test.

The F-statistic produced by each analysis was used to find the statistical probability that an sFFR response was present as a function of sweep count; a schematized overview of this approach is provided in Figure 1. Detection rate was quantified as the number of sFFRs detected by each statistical test in each listening condition. These proportions were compared using a Chi-square test. We calculated a “time-to-detect” measure as the test time, in seconds, at which detection probability was ≥99% and remained above this threshold for at least the following 25 sweeps. A repeated-measures analysis of variance (RMANOVA) was used to assess the effects of two factors (quiet vs. noise; F-test vs. HT^2^ test) on time-to-detect measurements.

## 3. Results

### 3.1. Detection Rate

Table 1 lists the percentage of participants from whom sFFR responses could be detected for each condition. Detection rate was similar across conditions [*X*(1) = 0.057, *p* = 0.811], demonstrating that comparable objective sFFR identification was achieved with either statistical approach in quiet or noise.

### 3.2. Time-to-Detect

**Figure 2:**
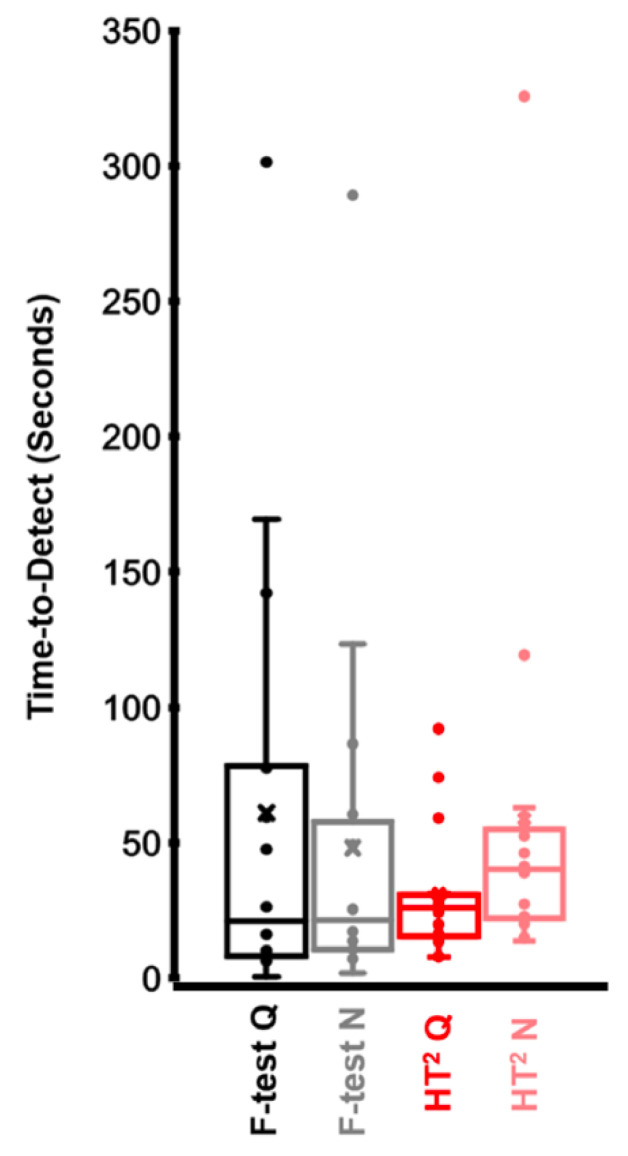
Time-to-detect by statistical test and condition. Each condition is coded with a different color for clarity. The middle line of the box represents the median, and the x represents the mean. The bottom line of the box represents the median of the 1st quartile, and the top line of the box represents the median of the 3rd quartile. The whiskers (vertical lines) extend to minimum and maximum values excluding outliers. Points that exceed 1.5 times of interquartile range are considered outliers. Outliers were not excluded from analyses because they represented the upper limit of test times for our sample.

Participants in which sFFR responses were not detected in any of the four conditions were removed from the time-to-detect analysis, leaving 16 participants. There was no main effect of statistical test (F-statistic vs. HT^2^ test; *F*_(1,15)_ = 1.475, *p* = 0.243, $\eta_{\rho }$^2^ = 0.09) or noise condition (quiet vs. noise; *F*_(1,15)_ = 0.172, *p =* 0.684, $\eta_{\rho }$^2^ = 0.011), nor was there an interaction between factors (*F*_(1,15)_ = 3.893, *p =* 0.067, $\eta_{\rho }$^2^ = 0.206). As shown in Figure 2, there was a trend for shorter averaged time-to-detect estimate with the HT^2^ test than with F-statistic in quiet, and there is less variability between participants when estimated with HT^2^ than F-statistic in both quiet and noise conditions.

## 4. Discussion

We predicted that the HT^2^ test, which utilizes more information contained in the sFFR spectrum than the F-test, would have superior detection performance than its counterpart for quiet and noise conditions. While there was a tendency for time-to-detect to be less variable when calculated from the HT^2^ test, we found no difference between statistical test performance in quiet or noise on our time-to-detect measure. These findings are broadly consistent with Easwar and colleagues (2020), who found that area under the curve metrics were similar between F- and HT^2^ tests. However, the same study indicated that the HT^2^ test had higher sensitivity than the F-test. Other reports have also shown time-to-detect advantages of the HT^2^ test for sFFRs measured in quiet, e.g., [6]. These discrepancies may be due to differences in sFFR stimuli between studies. For example, Vanheusden and colleagues (2019) used naturalistic monosyllabic words with a dynamic F0 and harmonics to evoke sFFRs, whereas the present study used a synthesized/da/stimulus with a stable F0 throughout the entire steady-state portion [6]. This likely resulted in robust F0 representation in the sFFR spectra; because this robust component was used in both F- and HT^2^ tests, performance of each test may have been more similar than predicted. We also expected that sFFR detection would be superior using the HT^2^ test in the noise condition. This prediction was based on the reasoning that noise is deleterious to sFFR spectral peaks, including the F0. Including more spectral peaks in the statistical test would therefore be expected to improve its ability to detect when a “true” sFFR is present. However, because we used speech-shaped noise as a masker, the sFFR components most impacted by the masker were the harmonics H2-H4 and not the F0. This is because the F0 component of the sFFR is dominated by neural phase-locking from higher frequency channels in which multiple harmonics overlap [18] and may not be appreciably affected by speech-shaped noise, which is low frequency dominant. This again would have made F- and HT^2^ test inputs more similar than expected and may have made their time-to-detect results more comparable in noise. To our knowledge, this is the first report to compare such sFFR detection methods in noise, and future studies with more realistic speech tokens and maskers are warranted.

This brief report adds to the growing literature demonstrating that statistically-based methods for automatic sFFR detection are robust, at least in listeners with normal hearing. Future parametric work is needed to explore the performance of these tests in listeners with hearing loss e.g., [11] or other audiologic disorders as well as the aging e.g., [26]. While objective detection methods are commonly used in commercial devices in which auditory steady-state responses can be obtained (e.g., Interacoustics, Intelligent Hearing Systems, GSI-Audera, Bio-logic), fewer proprietarry options exist for sFFR measurement and analyis and many clinician-scientists conduct analyses outside of commercially-availible systems (e.g., in MATLAB). This brief report and similar articles provide an evidence base for objective measures that can be employed in commercially available software for sFFR detection and analysis.

## Figures and Tables

**Table 1:** Percentage of response detection across conditions.

	Quiet	Noise
F-statistic	94% (17/18)	100% (18/18)
HT^2^	100% (18/18)	94% (17/18)

## Data Availability

Data used in this study will be provided without undue reservation upon contacting the senior author.
